# *In vivo* CRISPR editing for cancer immunotherapy

**DOI:** 10.3389/fimmu.2026.1872510

**Published:** 2026-07-29

**Authors:** Cole W. Christopher, Xiaoyu Zhou

**Affiliations:** 1 Vaccine and Immunotherapy Center, The Wistar Institute, Philadelphia, PA, United States; 2 Ellen and Ronald Caplan Cancer Center, The Wistar Institute, Philadelphia, PA, United States

**Keywords:** cancer, cell therapy, CRISPR, gene editing, gene therapy, immunotherapy, t cells, tumor microenvironment

## Abstract

Cancer immunotherapy has shown significant promise in certain patient populations, but further advancements are needed to extend its benefits to a wider range of patients. Clustered regularly interspaced short palindromic repeats (CRISPR)-based editing has rapidly evolved in recent years, enabling its transition into direct therapeutic applications. This review summarizes recent progress in applying CRISPR systems *in vivo* for cancer immunotherapy, focusing on approaches that target cancer cells and the tumor microenvironment, as well as those that directly engineer immune cell populations themselves. Novel CRISPR editing platforms and strategies enabling multiplexed editing have also recently demonstrated promising impacts on driving antitumor immunity, however, the platforms investigated are still in the early stages and further investigation will be needed to robustly assess the potential for clinical translation. Future work can expand the array of therapeutic targets by incorporating data from functional genomics and must also carefully evaluate both editing modalities and delivery systems to optimize efficacy, safety, and scalability.

## 1 Introduction

Immunotherapy has emerged as a promising therapeutic avenue for the treatment of various oncological malignancies. Immunotherapy broadly encompasses modalities that modulate the immune system to improve responses against cancerous cells and tissues (1). These modalities include immune checkpoint blockade (ICB), namely against PD-1 or CTLA-4 (2), cytokine therapy (3), cancer vaccines (4), T cell engagers (5), and engineered cell therapies such as chimeric antigen receptor (CAR) T cell therapy (6). Despite promising advances, many patients still do not respond to current immunotherapies, and new strategies are needed.

The advent of genome editing platforms, such as those based on clustered regularly interspaced short palindromic repeats (CRISPR)-Cas systems (7), has had substantial impacts on the immunotherapy field. These RNA-guided systems enable the targeted manipulation of DNA or RNA and can be adapted for a wide range of applications, including precise genome modification and programmable gene regulation. The rapid expansion of this toolkit has provided new opportunities to interrogate and engineer immune responses with increasing precision and flexibility (8). Studies have demonstrated, for example, that CRISPR editing can improve the efficacy of T cell therapies (9, 10). However, these investigations have largely focused on engineering immune cells *ex vivo* before transfer to the patient, which can be complex and costly. Recent advances in delivery platforms (11–13) have changed this paradigm by enabling direct *in vivo* genome editing (13–16), creating new opportunities to modulate both immune cells and cancer cells within their native context. This great potential could be further amplified through the incorporation of data from large *in vivo* CRISPR screens that directly evaluate the impacts of genetic perturbations on antitumor immune responses. These CRISPR screens will undoubtedly continue to play a substantial role in identifying actionable therapeutic targets.

**Figure 1:**
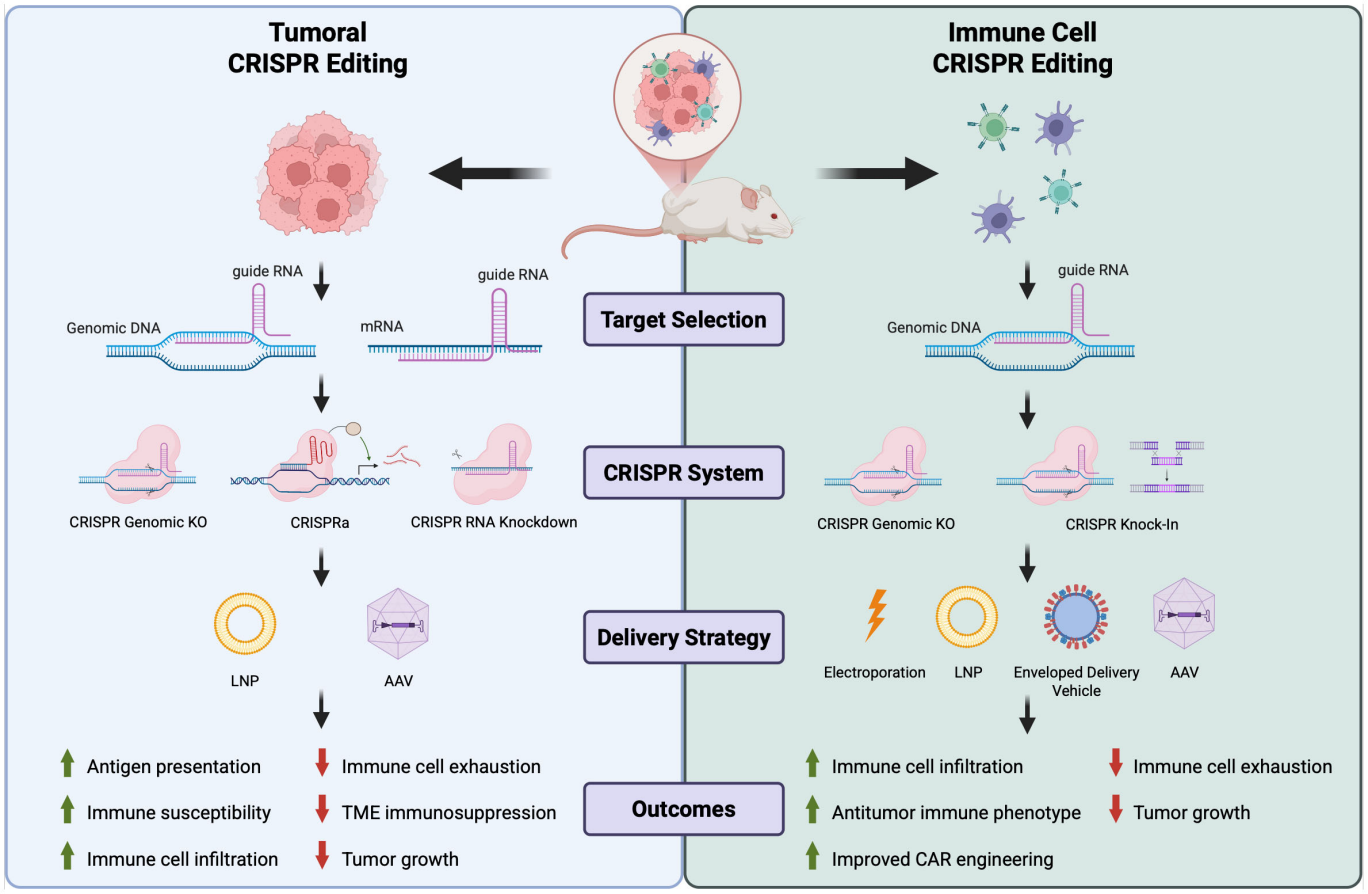
*In vivo* CRISPR editing for cancer immunotherapy. Schematic overview of two complementary approaches for *in vivo* CRISPR-based modulation of anti-tumor immunity: direct editing of tumors (left) and immune cell populations (right). Key considerations include target identification, choice of CRISPR editing platform (e.g., genomic knockout, knock-in, transcriptional regulation, or RNA targeting), delivery strategy (e.g., electroporation, LNP, enveloped delivery vehicles, or AAV). Tumor-directed editing aims to enhance antigen presentation, increase immune susceptibility, and promote immune cell infiltration while reducing tumor growth and immunosuppression within the tumor microenvironment. Immune cell-directed editing focuses on improving effector function, persistence, and infiltration, including applications such as CAR engineering. These strategies ultimately converge on enhancing antitumor immune responses while mitigating immune exhaustion and tumor progression. Created in BioRender. Christopher, C. (2026) https://BioRender.com/66urtl4.

Building upon these developments, *in vivo* CRISPR editing is emerging as a powerful strategy to augment antitumor responses without the need for complex *ex-vivo* manipulation. In this review, we focus on two general approaches to utilizing *in vivo* CRISPR editing for cancer immunotherapy: (i) editing tumor cells or the tumor microenvironment (TME) to enhance endogenous antitumor immunity, and (ii) direct engineering of immune cell populations, particularly T cells, to improve their antitumor function and/or persistence (**Figure 1**). While significant advances have also been made in related areas such as *in vivo* transgene delivery for the production CAR T cells, these topics have been thoroughly discussed elsewhere (17–19) and are not the focus of this review.

## 2 Framework for *in vivo* CRISPR therapeutics

### 2.1 CRISPR editing modalities

Multiple CRISPR-based modalities have been applied for *in vivo* use within the context of cancer immunotherapy. Genome editing systems using active nucleases, such as Cas9 and Cas12a, enable permanent gene knock-out or knock-in through DNA cleavage and repair, providing a means for the durable reprogramming of cellular function at the genomic level (8, 20, 21). However, the permanence of these modifications raises important safety considerations, particularly in the context of off-target effects (8, 22–24). More recently, base editing (25) and prime editing (26) systems have enabled precise sequence modifications without double-stranded breaks, potentially improving specificity and reducing genotoxic risk (8). In contrast, transcriptional modulation platforms, including CRISPR activation (CRISPRa) and interference (CRISPRi), allow for the reversible control of gene expression without altering the underlying DNA sequence (8, 27, 28), offering greater tunability. RNA-targeting systems such as Cas13 (29) further extend this spectrum by enabling transient knockdown at the RNA level, thereby avoiding permanent genomic changes (8). These modalities differ in durability, specificity, and risk profile, and their selection should be guided by the biological context and therapeutic objective. While multiple CRISPR editing platforms have been utilized in the context of cancer immunotherapy, most studies thus far have focused on more conventional Cas9-based gene knock-out systems. Future work can build upon these initial investigations, in part, by diversifying the utilized editing platforms, especially as novel editors continue to be rapidly developed as the field advances.

### 2.2 Delivery strategies

The clinical potential of *in vivo* CRISPR editing is fundamentally constrained by the ability to deliver editing components efficiently and specifically. This is the case for both tumor editing and immune cell editing strategies. Viral vectors, particularly adeno-associated viruses (AAV), have been widely used due to their high transduction efficiency (30). These AAV vectors have been utilized for the *in vivo* CRISPR editing of both cancer cells (31) and immune cells (32, 33). However, AAV vectors remain limited by their small packaging capacity, pre-existing immunity, dose-dependent toxicities, and the potential for rare vector genome integration, which together present important translational challenges (30). Non-viral platforms, most notably lipid nanoparticles (LNPs), have emerged as a versatile alternative, enabling the delivery of mRNA and ribonucleoprotein (RNP) complexes in a scalable and clinically tractable manner (34). LNP platforms have been utilized to deliver CRISPR editing components *in vivo* in both cancer cell editing (35–37) and immune cell editing (38, 39) contexts. More recently, engineered delivery systems, including targeted nanocarriers (40–43) and vesicle-based platforms (33, 44, 45), have been developed to enhance cell-type specificity (33, 42–45). Some delivery systems can also perform additional functions beyond cargo delivery (33, 37, 45, 46). For example, the recently described enveloped delivery vehicle (EDV) approach utilizes anti-CD3 and/or anti-CD28 single-chain variable fragments (scFvs) that confer T cell targeting capabilities while simultaneously activating the targeted T cells (33, 45). In addition, other emerging platforms for immune cell editing include *in vivo* electroporation systems with specialized devices (47).

Route of administration is an additional important consideration. In the context of tumoral CRISPR editing, many approaches have utilized intratumoral or local administration to deliver editing components directly to the tumor (31, 35–37, 41), but systemic approaches coupled with methods for promoting tumor-specific delivery have also been explored (40). Immune cell CRISPR editing platforms, on the other hand, largely necessitate utilizing systemic delivery approaches (32, 33, 38, 39, 44, 45) and so ensuring the platform can specifically and efficiently target the desired cellular population *in vivo* is of critical importance.

It is unclear which delivery strategy may have the most potential, and it is possible that different approaches may be appropriate depending on the disease context. For example, intratumoral delivery approaches may not be feasible for deep or difficult-to-access tumors, and so strategies utilizing systemic delivery may be required. Although many approaches have taken care to engineer delivery vehicles to promote cell-specific delivery, the potential for off-target editing cannot be discounted and should be a significant consideration when determining the potential for clinical translation. Future work could focus on comparing delivery strategies in a robust way to evaluate safety, specificity, and efficacy, as it is possible that a one-size-fits-all approach may not be appropriate for deciding the optimal delivery platform.

## 3 *In vivo* CRISPR editing for cancer immunotherapy

### 3.1 CRISPR engineering of tumors and the tumor microenvironment

Many strategies have been investigated for modulating the genome or transcriptome of cancer cells *in vivo* using CRISPR systems for immunotherapy (**Table 1**). CRISPR systems utilized to modulate tumors include genetic knockout approaches that use CRISPR-Cas9 systems to edit the cancer cell genome (31, 35–37, 40–43), as well as approaches that instead alter gene expression (48) or RNA transcript levels (49). These strategies focus on modulating tumor-intrinsic immunosuppressive factors, such as the upregulation of immune checkpoints, insufficient antigen presentation, and the release of immunosuppressive metabolites into the tumor microenvironment.

**Table 1:** *In vivo* editing of cancer cells for immunotherapy.

Cancer type	Genomic target	Editing platform	Delivery strategy	Outcomes	Ref
Bladder carcinoma	PD-L1, PCSK9	Cas9 KO	SWITCH (engineered nanoparticle)	Inhibition of tumor growth	(43)
Breast adenocarcinoma	Multiplexed	dCas9 (CRISPRa)	AAV	Enhanced antitumor immunity, increased T cell infiltration, increased APCs, increased TCR diversity	(48)
	Multiplexed; *Pdl1, Galectin9, Galectin3, Cd47*	Cas13d	AAV	Enhanced antitumor immunity, increased T cell infiltration, reduction in tumor growth, altered TME dynamics and cell populations	(49)
Colon	Multiplexed; *Pdl1, Galectin9, Galectin3, Cd47*	Cas13d	AAV	Enhanced antitumor immunity, increased T cell infiltration, reduction in tumor growth, altered TME dynamics and cell populations	(49)
Glioblastoma	PD-L1, CD47	Cas9 KO	LNP	Reduced tumor growth, enrichment of TAMs and T cells	(37)
Liver	PD-L1	Cas9 KO	LNP	Extension of survival, increased T cell and macrophage infiltration	(36)
Melanoma	PD-L1	Cas9 KO	Photoactivatable engineered nanoparticle	Inhibition of tumor growth, increased T cell infiltration, potentially altered cytokine expression in TME, potentially lower Treg frequency	(41, 42)
			Engineered nanoparticle	Induction of potent antitumor immunity, increased T cell infiltration, lower Treg frequency, altered cytokine expression in TME, increased DC maturation	(44)
	PD-L1, PCSK9	Cas9 KO	SWITCH (engineered nanoparticle)	Suppressed tumor growth, increased T cell infiltration, altered cytokine expression in TME	(43)
	LDHA	Cas9 KO	LNP	Suppressed tumor growth, increased T cell infiltration, increased T cell cytotoxicity	(35)
	Multiplexed; *Pdl1, Galectin9, Galectin3, Cd47*	Cas13d	AAV	Enhanced antitumor immunity, increased T cell infiltration, reduction in tumor growth, altered TME dynamics and cell populations	(49)
Ovarian	PD-L1	Cas9 KO	AAV	Extended survival, increased numbers of intratumoral T cells, fewer Tregs, altered cytokine expression in TME	(31)
			LNP	Inhibition of tumor growth, increased T cell infiltration, decrease of metastasis	(36)
Pancreatic carcinoma	PD-L1, PCSK9	Cas9 KO	SWITCH (engineered nanoparticle)	Inhibition of tumor growth	(43)
	Multiplexed; *Pdl1, Galectin9, Galectin3, Cd47*	Cas13d	AAV	Enhanced antitumor immunity, increased T cell infiltration, reduction in tumor growth, altered TME dynamics and cell populations	(49)

Table summarizing in vivo CRISPR editing strategies that have been recently investigated for cancer cell editing.

KO, knock-out; LNP, lipid nanoparticle; AAV, adeno-associated virus; APC, antigen presenting cell; TCR, T cell receptor; TME, tumor microenvironment; TAM, tumor-associated macrophage; DC, dendritic cell.

The number of CRISPR targets investigated in an *in vivo* context thus far is limited, with several studies specifically focusing on knockout of PD-L1 on cancer cells to abrogate well-established PD-1/PD-L1-mediated immunosuppression in the TME (31, 36, 37, 40–43). These studies establish proof-of-concept for directly editing immune evasion pathways *in vivo*, although their translational role in comparison to existing antibody-based checkpoint blockade therapies and current delivery limitations remain an area of ongoing investigation. A small number of other genomic targets have also been investigated for *in vivo* CRISPR-Cas9 knockout, including CD47 (37), PCSK9 (42), LDHA (35), and PTPN2 (43). These studies demonstrate the promise of *in vivo* CRISPR editing for promoting antitumor immunity through distinct mechanisms such as increased major histocompatibility complex class I (MHC-I) expression (42), decreased immunosuppressive metabolite levels in the TME (35), and restored interferon-gamma (IFN-γ) sensitivity (43). Collectively, these studies highlight the potential for utilizing CRISPR systems to modulate various pathways beyond the well-established immunosuppressive PD-1/PD-L1 axis.

Multiplexed CRISPR editing strategies have also been deployed for immunotherapeutic use. Indeed, one of the first examples of utilizing *in vivo* CRISPR systems for immunotherapy came from the multiplexed activation of endogenous genes as an immunotherapy (MAEGI) system developed by Wang et al. (48). This approach utilized AAV delivery of CRISPRa components *in vivo* to increase the presentation of mutant peptides in E0771 breast cancer cells and make tumors more susceptible to the immune system. This approach showed significant antitumor efficacy, particularly leading to increased T cell infiltration and an increase in MHC-II^+^ antigen-presenting cells (APCs), demonstrating the promise of utilizing CRISPR to augment anti-tumor immune responses directly *in vivo (*48). More recently, the same group developed multiplex universal combinatorial immunotherapy via gene silencing (MUCIG), utilizing Cas13d to silence multiple endogenous immunosuppressive genes through RNA knockdown (49). *In vivo* delivery of MUCIG vectors targeting inhibitory molecules including *PDl1, Galectin9, Galectin3*, and *Cd47* by intratumoral AAV injection demonstrated significant antitumor effects, coinciding with an increase in tumor-infiltrating lymphocytes (49). Together, these multiplexed approaches have the advantage of enabling the simultaneous targeting of multiple, orthogonal immunosuppressive pathways that limit antitumor immunity.

In general, *in vivo* CRISPR engineering of tumors shows significant promise for altering antitumor immune responses in the TME. Most notably, several approaches have demonstrated increased immune cell infiltration in tumors following gene editing, including by T cells (31, 35–37, 40–43) and macrophages (36, 37). Gene editing of tumors can also impact the expression of cytokines and cytotoxic effector molecules in the TME (31, 35, 40–43). Some studies have also demonstrated a decrease in regulatory T cells (Tregs) following tumoral gene editing (31, 41–43). These results collectively demonstrate how tumoral gene editing has immense potential for broadly modulating immune responses within the TME to promote antitumor immunity and improve outcomes. However, most studies have focused largely on evaluating tumor growth and immune cell infiltration, while the molecular and cellular mechanisms underlying these responses remain incompletely understood. Future studies should further dissect how CRISPR-mediated tumor editing reshapes the dynamic interactions within the TME to guide the rational design of more effective therapeutic strategies.

### 3.2 CRISPR engineering of immune cells

Another exciting recent advance has been the development of using targeted delivery strategies to directly edit immune cell populations themselves *in vivo* to enhance antitumor immune responses (**Table 2**). Much work has focused on optimizing delivery modalities to target the cell population of interest, which includes mainly T cells (32, 33, 38, 45, 47), but also macrophages (44) and dendritic cells (39).

**Table 2:** *In vivo* editing of immune cells for immunotherapy.

Immune cell type	Genomic target	Editing platform	Delivery strategy	Outcomes	Ref
T cells	PD-1	Cas9 KO	LNP	Reduced tumor growth	(38)
			Electroporation	Increased T cell infiltration, enhanced immune responses, increased antitumor immunity	(47)
	*Zc3h12a*	Cas9 KO	Ark313 AAV (+ Cas9-expressing mice)	Improved tumor control, increased T cell infiltration or proliferation	(32)
	*TRAC* (knock-in)	Cas9 KI (HDR)	EDV + AAV-hT7	Generation of enhanced, functional CAR T cells	(33)
Dendritic cells	PD-L1	Cas9 KO	LNP	Enrichment and maturation of cDCs, inhibition of tumor growth, increased activated T cells	(39)
Macrophages	*Pik3cg*	Cas9 KO	Nanovesicles	Reduced tumor growth, stabilization of antitumor M1 phenotype, altered TME from ‘cold’ to ‘hot’	(45)

Table summarizing in vivo CRISPR editing strategies that have been recently investigated for immune cell editing.

KO, knock-out; KI, knock-in; HDR, homology-directed repair; LNP, lipid nanoparticle; AAV, adeno-associated virus; EDV, enveloped delivery vehicle; CAR, chimeric antigen receptor; TME, tumor microenvironment; cDC, conventional dendritic cell.

For *in vivo* T cell editing, investigations have largely focused on well-established genomic targets such as PD-1. For example, recent studies have utilized *in vivo* CRISPR-Cas9 gene editing to knock-out PD-1 (38, 47) or *Zc3h12a (*32) in T cells to reduce exhaustion, increase proliferation and tumor infiltration, and overall improve antitumor responses. While these studies demonstrate important early advancements in *in vivo* immune cell CRISPR editing with promising outcomes, it is critical that more targets be investigated, including those that lay outside the scope of current immunotherapeutic strategies.

More recently, *in vivo* T cell CRISPR editing has also been utilized to generate improved CAR T cells through site-specific CAR transgene insertion (33). Specifically, Nyberg et al. demonstrated *in vivo* CAR T cell engineering by utilizing Cas9-mediated homology-directed repair (HDR) to insert a promoterless CAR transgene into the *TRAC* locus of T cells (33). This platform utilized CD3-targeted EDVs, which deliver CRISPR components specifically to T cells while also helping to induce T cell activation and proliferation (33). To enable efficient delivery of the HDR template to T cells *in vivo*, the authors coupled this approach with a novel evolved AAV, AAV-hT7, which demonstrated resistance to pre-existing neutralizing antibodies along with enhanced T cell tropism (33). By combining the CD3-targeting EDVs and AAV-hT7, the authors were able to knock-in a CAR transgene into the *TRAC* locus of T cells *in vivo*, leading to regulated and consistent CAR expression (33). These *in vivo*-generated CAR T cells demonstrated robust efficacy in both hematological and solid tumor mouse models, and additionally appeared to out-perform CAR T cells generated *in vivo* using conventional lentiviral vectors (33). This is an exciting advancement in *in vivo* CRISPR editing, as it broadly opens the door for the targeted insertion of various therapeutic transgenes directly *in vivo* with cell-type specificity. However, the approach still relies on AAV-mediated HDR template delivery and thus remains subject to the above-mentioned limitations of AAV vectors. Future development of non-viral donor delivery strategies and interrogation with emerging genome editing technologies that enable the insertion of large fragments, such as the PASTE (50), PASSIGE (51), and CAST (52) platforms, may further expand the efficiency, versatility, and clinical applicability of *in vivo* transgene insertion.

*In vivo* editing of other immune cells such as tumor-associated macrophages (TAMs) and dendritic cells has also been reported. One such example includes utilizing Cas9 RNP targeting *Pik3cg* while simultaneously delivering bacterial CpG-rich DNA fragments to stabilize a pro-inflammatory, anti-tumor M1 phenotype in TAMs (44). This approach also seemed to have impacts beyond TAMs, with beneficial effects on other immune cell populations such as T cells and dendritic cells as well (44). Additionally, knockout of PD-L1 in dendritic cells has also shown significant promise for promoting anti-tumor immunity, with broad impacts on the tumor microenvironment and associated immune cell populations (39). This included promoting a shift from pro-tumorigenic M2 macrophages to anti-tumor M1 macrophages within the TME while also increasing the infiltration of CD8^+^ T cells (39). *In vivo* PD-L1 knock-out in dendritic cells also seemed to promote dendritic cell activation and maturation (39).

Overall, these recent results demonstrate the promise of directly engineering immune cells *in vivo* to improve antitumor immunity and further show how the modulation of one cellular population can have more broad impacts on immune cell dynamics within the TME.

## 4 Discussion

*In vivo* CRISPR editing shows significant promise for cancer immunotherapy, particularly in overcoming the immunosuppressive tumor microenvironment. Strategies can involve either modulating cancer cells to increase susceptibility to immune responses or engineering immune cell populations to enhance potency. While both strategies demonstrate promise, their full potential likely lies in combinatorial approaches that target both cancer cells and immune cells.

Despite encouraging progress, several key limitations remain. The range of targets explored to date is relatively narrow, often centered on well-characterized immune regulatory pathways, limiting broader mechanistic insight. CRISPR-based strategies allow for the modulation of an incredibly wide array of genetic, epigenetic, and transcriptional targets, and current investigations have likely not explored the full therapeutic potential of this modality. In addition, delivery efficiency and specificity continue to constrain *in vivo* applications, particularly in systemic settings. A further challenge is the incomplete understanding of how gene perturbations in one cellular compartment propagate through the tumor-immune ecosystem, underscoring the need for more integrated and mechanistic studies.

Looking forward, several directions are likely to drive the next phase of development. Multiplexed editing strategies offer the opportunity to simultaneously target multiple, non-redundant immunosuppressive pathways. In parallel, transient editing approaches that do not lead to permanent genomic alterations (such as the MAEGI or MUCIG approach) may provide improved safety and tunability compared to permanent genome editing. Continued refinement of delivery systems will also be critical to enhance targeting specificity, tissue penetration, and overall editing efficiency. Ultimately, a systematic framework that integrates editing strategy, delivery considerations, and target cell biology will be essential for advancing *in vivo* CRISPR-based immunotherapies toward clinical translation.
